# Development of an Easy-To-Use Microfluidic System to Assess Dynamic Exposure to Mycotoxins in 3D Culture Models: Evaluation of Ochratoxin A and Patulin Cytotoxicity

**DOI:** 10.3390/foods13244167

**Published:** 2024-12-23

**Authors:** Veronica Zingales, Caterina Piunti, Sara Micheli, Elisa Cimetta, María-José Ruiz

**Affiliations:** 1 Research Group in Alternative Methods for Determining Toxics Effects and Risk Assessment of Contaminants and Mixtures (RiskTox), 46100 Valencia, Spain; m.jose.ruiz@uv.es; 2 Laboratory of Toxicology, Faculty of Pharmacy, University of Valencia, Av. Vicent Andrés Estellés s/n, 46100 Valencia, Spain; 3 Department of Industrial Engineering (DII), University of Padua, Via Marzolo 9, 35131 Padova, Italy; caterina.piunti@phd.unipd.it (C.P.); sara.micheli@unipd.it (S.M.); elisa.cimetta@unipd.it (E.C.); 4 Fondazione Istituto di Ricerca Pediatrica-Cittá Della Speranza (IRP-CdS)—Lab BIAMET, Corso Stati Uniti 4, 35127 Padova, Italy

**Keywords:** food contaminants, mycotoxins, cytotoxicity, in vitro models, 3D culture, neuroblastoma spheroid, microfluidic system

## Abstract

Mycotoxins are among the most concerning natural toxic food contaminants. Over the years, significant efforts have been made to characterize the risk associated with their exposure. However, assessing their toxicity has so far been elusive due to the lack of adequate models that closely mimic the physiological conditions of human cells *in vivo*. Here, we present the SpheroFlow Device (SFD), an efficient microfluidic platform designed, manufactured, and validated to evaluate mycotoxin-induced cytotoxicity under dynamic and continuous exposure in 3D culture settings. In the present study, we integrated human neuroblastoma SH-SY5Y spheroids into the SFD to assess the acute toxicity induced by the mycotoxins ochratoxin A (OTA) and patulin (PAT). The developed system enabled qualitative and quantitative measurements of concentration–response relationships and provided accurate control over the culture microenvironment. Our findings show that by enhancing 3D culture model by applying dynamic flow, SH-SY5Y spheroids exhibited different sensitivities to OTA and PAT compared to conventional static SH-SY5Y spheroids, confirming the critical role of culture models in mycotoxin toxicity assessment. This is the first study assessing the neurotoxicity of OTA and PAT on 3D neuroblastoma spheroids considering the contribution of fluid flow.

## 1. Introduction

For decades, animal and standard *in vitro* culture models have proven invaluable in advancing the knowledge of the effects induced by the exposure to toxicants. However, these approaches are affected by ethical issues and several limitations [1,2,3]. In response to the pressing need for more predictive and translationally relevant methods, technological advances in bio- and micro-fabrication are revolutionizing toxicological research by providing tools that more closely recapitulate the complex architecture and environment of native target organs. To do so, the scientific community is increasingly looking at three-dimensional (3D) cell cultures that allow cells to self-organize and grow omni-directionally. When combined with perfusion flows, the performance of 3D cultures is further improved by simulating a physiological environmental condition and mechanical cues comparable with the human circulation [4]. In parallel, the integration of 3D models with microfluidic technologies enables improved control of the spatial and temporal dynamics of *in vivo* microenvironments [5]. Encouraging evidence suggests that the field of food toxicology is poised to benefit from advanced alternative methods, as they not only align with the ethical imperative and regulatory pressure advocated by the Food and Drugs Administration (FDA) and the European Food Safety Authority (EFSA) but also represent a significant step forward in ensuring food safety by offering greater precision, efficiency, and relevance to human health [6].

Food is a potential reservoir of chemical toxic contaminants, which pose serious economic and health threats. Among the most common food contaminants, mycotoxins are particularly critical, which often lead to food and feed border rejections, economic losses, and significant adverse effects on human and animal health [7]. Mycotoxins are small secondary metabolites produced by filamentous fungi that can colonize crops both in the field and during storage. Among the mycotoxins of primary concern due to their toxicity and prevalence are ochratoxin A (OTA) and patulin (PAT). OTA is produced by species belonging to the genera *Aspergillus* (e.g., *A. ochraceus*) and *Penicillium* (e.g., *P. verrucosum*) [8]. It is a ubiquitous mycotoxin that has been extensively documented in a wide range of foods, including cereals and cereal-based products, grapes, wine, dried fruits, spices, and green coffee [9,10,11]. OTA is a nephrotoxin with carcinogenic potential for humans (Group 2B, based on the International Agency for Research on Cancer classification (IARC) [12,13,14]. Additionally, it exhibits hepatotoxicity [15,16], neurotoxicity [17], genotoxicity [18,19], and immunotoxicity [20,21], though these effects are less well characterized [22]. PAT, mainly produced by *P. expansum*, is mostly detected in moldy fruits and fruit-based products [23]. Currently, the IARC classifies PAT as Group 3 (unclassifiable regarding its carcinogenicity in humans) [24]; however, studies on its toxicity have associated it with adverse immunological [25,26], gastrointestinal [27,28,29,30], and neurological outcomes [31,32,33]. As such, the full implications of OTA and PAT exposure for human health remain poorly understood.

To date, despite significant efforts to overcome the limitations of traditional toxicology and advance towards the adoption of human-relevant new approach methodologies (NAMs), few studies have employed alternative methods to assess mycotoxin toxicity [34]. Among mycotoxins, aflatoxin B1 (AFB1) is the most extensively studied using alternative *in vitro* models. Its genotoxic potential has been explored through the use of mono-type and co-culture spheroids, as well as microphysiological systems (MPSs) that enable the preservation of hepatocyte functionality [35,36,37,38,39,40,41]. For other mycotoxins of concern, although studies employing 3D culture models are becoming more common, the use of organ-on-a-chip systems remains limited [42,43,44,45,46]. Regarding OTA and PAT, to the best of our knowledge, no study has yet employed organ-on-a-chip models to assess PAT toxicity, while for OTA, only one study has used a 3D human kidney proximal tube MPS to define the dose–response relationships of OTA-induced nephropathy, highlighting its potential as a valuable tool to reflect chronic OTA toxicity [47].

In the present study, we designed, manufactured, and validated the SpheroFlow Device (SFD), an efficient microfluidic platform dedicated to defining mycotoxin-induced cytotoxicity under dynamic and continuous exposure in 3D culture settings. Human neuroblastoma SH-SY5Y spheroids were used as a target model, since exposure to both mycotoxins has been associated with neurotoxicity, but only limited studies assessed their effects on the neuronal system. The device enabled qualitative and quantitative measurements of dose–response relationships and accurate control over the culture microenvironment with relative simplicity. Techniques and methodologies were optimized to obtain a reliable and repeatable tool that matches high-throughput requirements, easy and effective reading, and data analyses.

## 2. Materials and Methods

### 2.1. Reagents

The reagent-grade chemicals and cell culture compounds listed below were purchased from Gibco (Paisley, UK): DMEM high glucose culture medium with L-glutamine, fetal bovine serum (FBS), minimum essential medium nonessential amino acids (MEM NEAA), and phosphate-buffered saline (PBS). Penicillin, streptomycin, and trypsin/EDTA solutions were obtained from Corning (Rochester, NY, USA). Standards of the selected mycotoxins, namely OTA (MW: 403.81 g/mol) and PAT (MW: 154.12 g/mol), as well as acetone, propylene glycol monomethyl ether acetate (PGMEA), isopropanol, methanol (MeOH), Thiazolyl Blue Tetrazolium Bromide (MTT), *t*-octylphenoxypolyethoxyethanol (Triton-X 100), dimethyl sulfoxide (DMSO), and Hoechst 33342, were purchased from Sigma-Aldrich (St. Louis, MO, USA). The LIVE/DEAD™ Viability/Cytotoxicity Kit was from Thermo Fisher Scientific (Waltham, MA, USA). Stock solutions of the mycotoxins were prepared in MeOH and maintained at +4 °C for OTA and at −20 °C for PAT.

### 2.2. Cell Culture and Spheroid Formation

SH-SY5Y (ATCC CRL-2266) cells were maintained in culture in monolayer in DMEM high glucose with L-glutamine medium supplemented with 10% FBS, 1% MEM NEAA (100×) and 1% penicillin/streptomycin under standard conditions (37 °C and 5% CO_2_ humidified atmosphere). The medium was changed every 2–3 days. Spheroids were generated from trypsinized monolayer cells, as previously described [48,49]. Briefly, single-cell suspensions were diluted to 1 × 10^4^ cells/mL density. Then, 200 μL of cell suspension (2 × 10^3^ cells) was dispensed into each well of an Ultra-Low Attachment (ULA) 96-well round bottom plate (Corning^®^, New York, NY, USA) and centrifuged at 1200 revolution per minute (RPM) for 10 min to aid in the self-assembly of cells at the bottom of the wells. The spheroids were cultured for 7 days, with a gentle 50% medium replenishment on day 4.

### 2.3. Design and Production of the SFD

**Figure 1:**
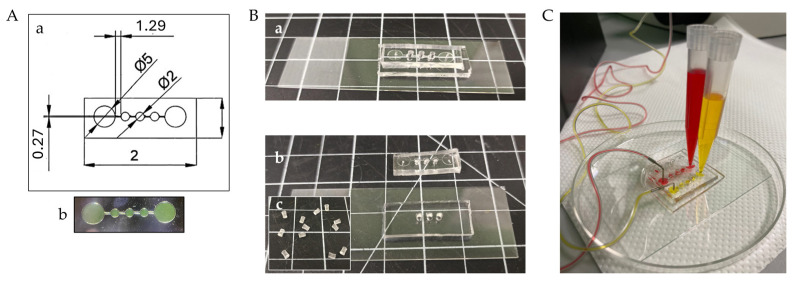
SpheroFlow Device (SFD) design, fabrication, and validation. (**A**) Device design and mold fabrication: (**A-a**) The device was designed using AutoCAD^®^. Dimensions in mm. (**A-b**) Detail of the master mold produced via photolithography. (**B**) Microfluidic device assembly: (**B-a**) Microfluidic device in its final configuration and its individual parts: (**B-b**) upper layer, bottom layer sealed to a glass slide by plasma treatment (**B-c**) PDMS plugs. (**C**) Representative image of the fluid dynamic validation with flowing color tracers.

The design of the SFD was realized using the AutoCAD^®^ software (https://web.autocad.com/) (Figure 1A). The device comprised an inlet and an outlet reservoir (diameter 5 mm) and three culture wells (diameter 2 mm) connected through a microchannel (0.27 × 1.29 mm width × length). Starting from the designed geometry, a negative photomask was generated using Adobe Illustrator^®^ (Figure S1). The photomask was used to produce the master mold via photolithography, as previously described by Angiolillo et al. [50], with some modifications. In detail, a 10 cm silicon wafer was cleaned with acetone, MeOH, and distilled water, followed by drying with compressed air. The wafer was then placed inside a desiccator saturated with hexamethyldisilazide vapors (Sigma-Aldrich, 440191) for 15 min to promote photoresist adhesion. The wafer was inserted in a spin coater (WS-650-23B, Laurell Technologies Corporation, Lansdale, PA, USA) to deposit a layer of SU-8 2100 negative photoresist (Microchem, Fiorenzuola, Italy), obtaining a final thickness of 250 μm. After the soft bake heat treatment at 95 °C for 50 min, the wafer was covered with the photomask and exposed to UV light (350 mJ/cm^2^ for 120 s), selectively cross-linking the regions corresponding to the transparent areas. Post-exposure baking at 95 °C for 15 min completed the cross-linking. Development was carried out using Propylene glycol monomethyl ether acetate (PGMEA) for 17 min, followed by rinsing with isopropanol and drying with compressed air. A hard bake was performed by heating the wafer in an oven at 100 °C for at least 1 h.

Polydimethylsiloxane (PDMS, Sylgard^®^ 184, Dow Corning, Midland, MI, USA) was used for replica molding as described by Micheli et al. [51]. Briefly, PDMS was prepared by mixing the base and the curing agent in a 10:1 *w*/*w* ratio, degassed to remove all air bubbles, and poured on the silicon mold. After baking at 80 °C for 45 min, the polymerized PDMS slab was peeled from the mold and punched in correspondence to the inlet and the outlet with a 1 mm stainless steel biopsy punch (Nordson EFD, East Providence, RI, USA) and with a 2 mm punch to create the 3 culture wells for the spheroids.

This PDMS layer was bound via plasma treatment to an additional PDMS layer containing three 2 mm punched holes aligned with the culture wells of the upper layer. After bonding, the double-layer PDMS structure was sealed onto a glass slide with plasma treatment. The assembled device was sterilized via autoclaving before use.

### 2.4. SFD Validation and Spheroids Integration

#### 2.4.1. Fluid Dynamic Validation

The assembled SFD was validated in terms of hydraulic sealing with colored dyes injected using a syringe pump (PHD Ultra, Harvard Apparatus, Cambridge, MA, USA). Briefly, syringes were filled with colored solutions, fixed to the pump, and connected by silicone tubes to the inlet of the device. The outlets were connected to 1 mL tips to complete the configuration and collect the outflowing dye. PDMS plugs were used to prevent any leakage from the culture wells. The pump was configured to operate at a flow rate of 1 μL/min, and the device was continuously perfused for 24 h.

#### 2.4.2. Spheroid Integration in the SFD and Biological Validation

To prevent adhesion of the SH-SY5Y spheroids to the bottom of the device, a solution of 1% of pluronic (Sigma Aldrich, St. Louis, MO, USA) in PBS was used to coat the device, as previously described [52]. The coating solution was injected through the inlet of the platform and left overnight at room temperature. After that, a washing and a de-bubbling step were performed using milliQ water. Finally, 7-day-old SH-SY5Y spheroids were transferred from the ULA 96-well growth plate to the SFD (one spheroid per culture well) using a micropipette with a cut tip. The culture wells were then sealed with PDMS plugs, while syringes filled with culture medium were fixed to the pump and connected by silicone tubes to the inlet of the microfluidic device. The spheroids were subjected to a flow rate of 1 µL/min of culture medium for 24 h, and at the end of the selected time point, cell viability was assessed using the LIVE/DEAD assay (see Section 2.6.1).

### 2.5. Mycotoxin Treatment

Concentration–response relationships of OTA and PAT toxicity in the SFD-integrated SH-SY5Y spheroids were assessed with increasing concentrations of OTA (from 0 to 100 µM) and PAT (from 0 to 12.5 µM). Briefly, after seeding the spheroids into the SFD as previously described (see Section 2.4.2 for details), syringes were filled with medium containing OTA or PAT at the desired concentration, fixed to the PHD Ultra pump, and connected by tubes to the inlets of the microfluidic device. Subsequently, the chip was run for 24 h at a flow rate of 1 µL/min. Six serial concentrations were tested for each mycotoxin with a dilution factor of 2. The concentrations were selected considering our previous study assessing the individual toxicity of mycotoxins on spheroids under static conditions [48]. Specifically, the concentration ranges for OTA and PAT were determined based on their reported occurrence in food and the half maximal inhibitory concentrations (IC_50_) available in the literature [53]. In addition, these ranges were adjusted to account for the typically lower sensitivity observed in 3D cultures compared to monolayer cells [54,55]. For each experiment, a solvent control (syringes filled with medium containing the same amount of MeOH) and a positive control (syringes filled with medium containing 20 μL/mL of 1% Triton-X) were included. The SFD configuration, designed to accommodate three spheroids, ensured that each spheroid within the chip was simultaneously exposed to the same condition, allowing each condition to be tested in triplicate. Additionally, three independent experiments (each with three technical replicates) were carried out for all conditions tested.

### 2.6. Measurement of Cell Viability

#### 2.6.1. LIVE/DEAD Assay

Calcein-AM (Invitrogen^®^, C3100MP, Carlsbad, CA, USA), propidium iodide (Sigma-Aldrich, P4864) and Hoechst 33342 were used to assess the spheroids’ viability, according to Angiolillo et al. [50], with some modifications. Calcein-AM is a non-fluorescent, cell-permeant compound that is converted into a fluorescent molecule (green fluorescence 495–515 nm) by the enzymatic activity of esterases found in viable cells, while propidium iodide is a nucleic acid-intercalating agent that is internalized by cells with damaged membranes, leading to enhanced fluorescence signal (red fluorescence 495–635 nm) upon DNA binding. The staining solution was prepared by diluting 1 mM Hoechst 33342, 1 mM Calcein-AM and 1.5 mM propidium iodide in PBS at ratios of 1:500, 1:500, and 1:250, respectively. Briefly, medium from the SFDs was discarded through three washes with PBS. Afterwards, the staining solution was injected through the inlet of the platform and then incubated for 30 min at 37 °C. After the staining procedure, the chips were imaged using a confocal fluorescence microscope (ZEISS LSM 800 Airyscan, Zeiss Microscopy, Jena, Germany) to visualize live (green-stained cells) and dead cells (red-stained nuclei) with nucleus marker Hoechst 33342 (blue-stained cells). For each spheroid, a *z*-stack of 10 slices was acquired to capture the information derived from the three-dimensionality of the structure. After acquisition, the images were processed using the orthogonal projection method.

#### 2.6.2. MTT Assay

The cytotoxicity of OTA and PAT on SH-SY5Y spheroids after 24 h of dynamic exposure was determined quantitatively using the MTT assay. This method is based on the ability of viable cells to metabolize the yellow soluble tetrazolium salt to a blue insoluble formazan product by the mitochondrial succinic dehydrogenase. The MTT assay was performed as previously described [49], with slight modifications. In short, at the selected time point, single spheroids were transferred to an empty 96-well flat bottom plate with 100 µL/well of fresh medium and 50 μL/well of MTT solution (5 mg/mL PBS). After 4 h of incubation at 37 °C protected from light, the resulting formazan crystals were solubilized in DMSO (50 μL/well). The absorbance was measured at 560 nm using Spark^®^ Multimode Microplate Reader by Tecan (Männedorf, Switzerland). Cell viability was expressed as a percentage relative to the solvent control (MeOH). The IC_50_ values were calculated using Graphpad Prism version 8.0.2 (nonlinear regression (curve fit) [Inhibitor] vs. normalized response; GraphPad Software, San Diego, CA, USA).

### 2.7. Statistical Analysis

Statistical analysis was carried out using GraphPad Prism version 8.0.2 (GraphPad Software, San Diego, CA, USA), statistical software package [56]. Data were expressed as mean ± SEM of three different independent experiments. A 2-tailed Student’s *t*-test was performed to assess the difference between paired samples. We set a significance level of 0.05, such that *p* ≤ 0.05 was considered statistically significant.

## 3. Results

### 3.1. Design and Fluid Dynamic Validation of the SFD

The platform was designed with three interconnected culture wells, each housing a single spheroid. The SFD was compatible with the dimensions of a standard microscope slide, occupying an area of approximately 25 × 8.3 mm^2^, with a height of 250 μm (Figure 1A). The final configuration consisted in an upper layer containing the device geometry and a bottom PDMS layer designed to increase the culture volume for the spheroids; PDMS plugs were obtained to seal the culture wells and ensuring hydraulic integrity (Figure 1B). Before biological integration, the device was validated from the fluid dynamic standpoint. To verify that the plasma treatment properly activated all surfaces and that all parts of the system adhered correctly to each other to create a closed hydraulic circuit, colored solutions were injected through the inlet of the platform. As shown in Figure 1C, the colored solutions properly filled all microchannels, and no dye leakage was observed between the two layers of the chip and around the wells covered by the plugs, indicating that the hydraulic system was properly and irreversibly sealed.

### 3.2. Biological Validation of the SFD

**Figure 2:**
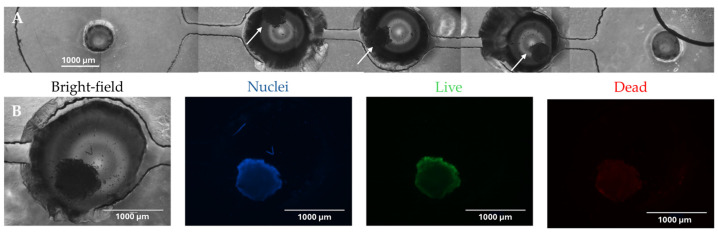
Biological validation of the SFD. (**A**) Reconstruction of the entire length of the device including the inlet, culture wells, and outlet regions after 24 h of perfusion. Arrows indicate the spheroids. Scale bar: 1000 µm. (**B**) Cell viability (LIVE/DEAD assay) evaluated after 24 h of flow. From left to right: representative brightfield and fluorescent images of SH-SY5Y spheroids stained with Hoechst (nuclei, in blue), Calcein-AM (live cells, in green), and propidium iodide (dead cells, in red). Scale bar 1000 µm. Images were obtained using the fluorescence microscope EVOS™ FL at 4× magnification (Invitrogen, Carlsbad, CA, USA).

The structural stability of the SH-SY5Y spheroids integrated into the SFD, as well as the perfusion conditions (flow rate: 1 µL/min for at least 24 h) needed to maintain viable spheroids, were also evaluated. Figure 2A shows the spheroids after being subjected to a flow rate of 1 µL/min for 24 h. The 3D constructs retained their original shape and position within the culture wells under the applied perfusion forces. Cell viability was assessed using the LIVE/DEAD assay (Figure 2B), where the bright green signal correlated with live cells, while the slight red signal indicated low numbers of propidium iodide-stained dead cells. These results confirmed the biocompatibility of our microfluidic platform and its ability to effectively integrate spheroids.

### 3.3. Assessment of OTA Toxicity Using the SFD

**Figure 3:**
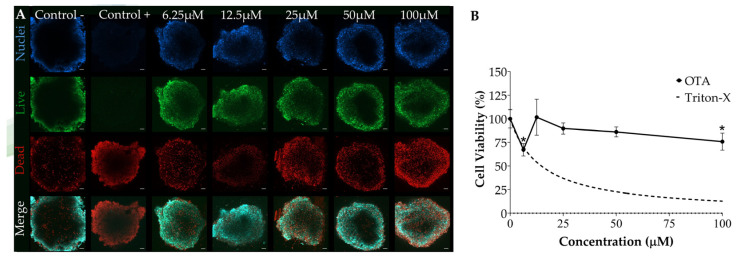
Concentration–response relationship between OTA toxicity in SH-SY5Y spheroids integrated into the SFD. SH-SY5Y spheroids were exposed to increasing concentrations of OTA for 24 h at a flow rate of 1 µL/min. Cell viability was assessed via (**A**) LIVE/DEAD assay and (**B**) MTT assay. (**A**) Representative fluorescent images of spheroids stained with Hoechst (nuclei, in blue), Calcein-AM (live cells, in green) and propidium iodide (dead cells, in red). Images were obtained using a confocal fluorescence microscope (ZEISS LSM 800 Airyscan, Zeiss Microscopy, Germany), acquiring a z-stack of 10 slices for each spheroid to capture the information derived from the three-dimensionality of the structure. Images were processed using the orthogonal projection method. Scale bar: 50 µm (objective 20×). (**B**) Concentration–response curve from MTT data. Data are expressed as mean ± SEM of three independent experiments (*n* = 3). (*) *p* ≤ 0.05 indicates a significant difference compared to the control. Triton-X was used as a positive control.

The short-term effects of OTA on SH-SY5Y spheroids integrated into the SFD were investigated using LIVE/DEAD and MTT assay after 24 h of exposure (Figure 3). Our results revealed a toxicity induced by OTA exposure, as evidenced by the drastic increase in dead cells observed in Figure 3A. Interestingly, a concentration-dependent toxicity pattern was detected starting from 12.5 µM, which exhibited a lower cytotoxicity compared to the lower concentration 6.25 µM. These qualitative results were confirmed by the MTT assay (Figure 3B), which showed a significant reduction in cell viability at both the lowest (6.25 µM) and the highest (100 µM) concentrations tested. In contrast, at 12.5 µM, cell viability remained comparable to solvent control levels (% cell viability = 101.69 ± 19.00). Therefore, by comparing the two assays, it can be concluded that OTA induces a progressive increase in cell mortality within the concentration range of 12.5–100 µM, with statistical significance confirmed only at 100 µM by the MTT test. Notably, both assays highlight a lower cytotoxic effect of OTA at the concentration of 12.5 µM compared to 6.25 µM. Furthermore, based on our MTT assay results, no IC_50_ value was reached within the range of concentrations tested.

### 3.4. Assessment of PAT Toxicity Using the SFD

**Figure 4:**
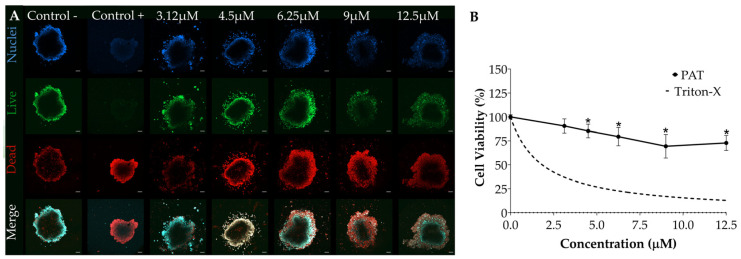
Concentration–response relationship of PAT toxicity in SH-SY5Y spheroids integrated into the SFD. SH-SY5Y spheroids were exposed to increasing concentrations of PAT for 24 h at a flow rate of 1 µL/min. Cell viability was assessed via (**A**) LIVE/DEAD assay and (**B**) MTT assay. (**A**) Representative fluorescent images of spheroids stained with Hoechst (nuclei, in blue), Calcein-AM (live cells, in green) and propidium iodide (dead cells, in red). Images were obtained using a confocal fluorescence microscope (ZEISS LSM 800 Airyscan, Zeiss Microscopy, Germany), acquiring a z-stack of 10 slices for each spheroid to capture the information derived from the three-dimensionality of the structure. Images were processed using the orthogonal projection method. Scale bar 100 µm (objective 10×). Panel (**B**) Concentration–response curve from MTT data. Data are expressed as mean ± SEM of three independent experiments (*n* = 3). (*) *p* ≤ 0.05 indicates a significant difference compared to the control. Triton-X was used as a positive control.

Similarly, to determine the acute cytotoxicity of PAT under dynamic conditions, we exposed SH-SY5Y spheroids integrated into our SFD to increasing concentrations of mycotoxin. After 24 h, the spheroids showed signs of severe cytotoxicity, with a relevant increase in dead cells as PAT concentrations increased (Figure 4A). The MTT assay (Figure 4B) again confirmed these findings, highlighting significant cytotoxicity induced by all tested concentrations, except for the lowest one (3.12 µM), and a concentration-dependent decrease of cell viability in the concentration range of 4.5–9 µM (Pearson correlation coefficient = −0.93). Similar to OTA, also for PAT, the IC_50_ value was not reached under the tested conditions.

## 4. Discussion

We here provided evidence that the SFD we developed is a promising tool for the investigation of mycotoxin-mediated toxicity in a more physiological way. Although bioengineering strategies are becoming widespread in the cell culture space, they are less frequently applied in the fields of food toxicology and mycotoxin studies. The integration of advanced bioengineering techniques, such as 3D cell cultures, organ-on-a-chip models, and dynamic culture systems, holds significant potential to better mimic the *complex in vivo* environment and provide more realistic insights into the effects of mycotoxins on human health, ultimately enhancing food safety and public health outcomes. Unfortunately, the adoption of these technologies in food toxicology has been limited, likely due to the challenges associated with transitioning from traditional methods to more sophisticated advanced models [34]. In this study, we present a simple and intuitive microfluidic platform that is capable of providing the critical items needed to overcome the main challenges associated with most of the complex advanced alternative systems already described in the literature. Our device is practical to use; it features a simple design, low fabrication complexity, and cost-effective manufacturing process, while also ensuring high throughput and reproducibility. Multiple platforms can be run simultaneously, allowing for different experimental conditions (one for each device) with a substantial number of technical replicates.

Our SFD offers a new approach to assess the potential toxicity of various compounds, including mycotoxins, being able to combine a physiologically relevant 3D culture model with microfluidic-perfused conditions. Microfluidic devices have convincingly demonstrated that fluid flow is a crucial trigger for many aspects of human physiological complexity. The flowing medium, comparable to human circulation, mimics the continuous supply of oxygen and nutrients, mirrors the delivery of drugs or other compounds of interest to the cells, and enables the generation of mechanical cues [57]. Furthermore, by using the SFD, it was possible to enhance standard 3D culture performance and define a more realistic *in vitro* model with a precise control of the microenvironment. The device and perfusion flow set displayed excellent biocompatibility with our 3D culture model and allowed for a continuous mycotoxin-containing medium supply at a constant flow rate without exposing the cells to a high shear stress. The use of Triton-X as a positive control, then, assisted in evaluating the functionality and performance of the model.

We here integrated neuroblastoma SH-SY5Y spheroids into the SFD to evaluate the toxicity induced by acute exposure to OTA and PAT. Building on the results of our previous studies that proved the importance of the culture models in mycotoxin cytotoxicity screening [48,49], we here provided evidence of a different sensitivity of SH-SY5Y spheroids exposed to mycotoxins under dynamic conditions. Based on our findings, the LIVE/DEAD assay revealed a notable presence of dead cells after exposure to both mycotoxins. Quantitative analysis of cell viability through the MTT assay indicated a relatively slight but significant decrease in cell viability in both cases, with peaks of mortality of 33% for OTA and 31% for PAT. Compared to our previously reported toxicity data on static 2D and 3D spheroids (summarized in Table 1), the IC_50_ value for OTA in SFD-integrated SH-SY5Y spheroids was in good agreement with that obtained in SH-SY5Y spheroids under static conditions. Indeed, in both exposure conditions, the IC_50_ was not reached within the range of concentrations tested. However, a different trend in viability can be observed between the two culture models. Specifically, continuous and dynamic exposure to OTA for 24 h resulted in an effect at both the highest and lowest concentrations tested. In contrast, our previous study under static conditions showed no significant decrease in viability across all concentrations (6.25–100 µM) [48]. Additionally, the response of SH-SY5Y spheroids to continuous and dynamic exposure to OTA demonstrated a pattern characterized by cell death stimulation at low concentrations and inhibition at higher ones, suggesting an adaptive response to high doses of the stressor [58,59]. Therefore, the concentration–response curve obtained under dynamic exposure suggest a biphasic dose response, which was not appreciated under static conditions [48]. An even more marked difference in sensitivity to OTA depending on the exposure mode was also demonstrated in kidney spheroids. In particular, the kidney MPS showed an IC_50_ value of 1.21 µM after 72 h of exposure [47], indicating less sensitivity compared to static kidney spheroids, which had an IC_50_ value of 1.7 µM after 24 h of exposure [42].

Regarding PAT, in our dynamic model its cytotoxic effects tend to be weaker and start at higher concentrations compared to conventional static 3D SH-SY5Y spheroids [48]. As a matter of fact, while in the present study, no IC_50_ value was reached, under static conditions, we previously obtained an IC_50_ value of 4.93 µM (Table 1). The different sensitivity between static and dynamic conditions could be attributed to the effects of the flow. Specifically, by shedding light on the variability in IC_50_ values and cell viability behavior obtained in SH-SY5Y spheroids in response to OTA and PAT depending on the exposure conditions, these findings raise serious concerns about the potential mismatch between the actual risk scenario for humans due to mycotoxins exposure and that predicted by current toxicity assessments. And, this discrepancy largely stems from the absence of a standardized and integrative study strategy. Since a dynamic environment is known to promote improved maintenance of cell structures and modulate cell responsiveness to external stimuli, incorporating dynamic factors into experimental models is expected to significantly enhance their performance and predictive accuracy [60,61,62]. Our proposed approach aims to better recapitulate the complexity of living systems by offering a more robust and reliable framework for toxicity prediction. This study has sought to pave the way for the importance of selecting an appropriate model for assessing mycotoxin toxicity, emphasizing that the culture model can significantly influence cytotoxicity outcomes. It also highlights the critical need to conduct cytotoxicity screenings in advanced, *in vivo*-like culture systems that better reflect human physiology. Such models are essential to accurately estimate the risks associated with mycotoxin exposure and to advance our understanding of their toxicological impact.

Overall, we demonstrated that the platform described here is suitable for the assessment of mycotoxin toxicity. To the best of our knowledge, this is the first study assessing the cytotoxicity of OTA and PAT on a 3D neuroblastoma spheroids-on-a-chip model, and it is the first to consider the contribution of flow and shear stress to their neurotoxicity. Driven by the need to improve knowledge on the neurotoxic effects of OTA and PAT, our results show the effects of these mycotoxins exclusively on neuroblastoma spheroids. Nonetheless, we are aware of the complexity of the human body, which consists of several cell types and interconnected players. Fully aware of the limitations of our model, we selected this culture model because it had already been well-characterized and provided a solid foundation for comparing OTA and PAT toxicity under both static and dynamic conditions. In future applications, the use of the SFD can, of course, be extended to other cell lines or even multicellular organoids; as well, it can be further adapted to integrate multiple organs. Indeed, the inherent versatility of the SFD, stemming from its protocol of transferring pre-formed spheroids from ULA plates, stands out as one of its main advantages. However, to ensure optimal performance and reproducibility, it is essential to develop and implement cell type-specific fluid flow rate protocols.

## 5. Conclusions

In this study, we provided evidence that by further improving our 3D culture model by applying a dynamic flow, SH-SY5Y spheroids demonstrated different sensitivities to OTA and PAT. Therefore, building on our previous results and on the evidence that MPS are more physiological than static cultures, we here further confirm the importance of the culture model in mycotoxin toxicity assessment. This *in vitro* platform can be further developed to become an ideal tool to assess mycotoxin-induced cytotoxicity, even at longer exposure times, and supports changes in mycotoxin risk assessment in a more realistic exposure scenario. The increased application of bioengineering strategies in the field of food toxicology, such as the use of this device requiring minimal personnel training, aligns with the 3R principles of animal replacing, reducing, and refining and will undoubtedly improve the predictability of current *in vitro* assays.

## Figures and Tables

**Table 1:** The half maximum inhibitory concentration (IC_50_) values of OTA and PAT measured in SH-SY5Y cells cultured as monolayers (2D) and spheroids in static conditions (3D) [data from our previous study [48], and spheroids in dynamic conditions inside the SFD (3D-SFD). Data are expressed as the mean ± SEM of three independent experiments (*n* = 3).

IC_50_ Value (μM) ± SEM			
Mycotoxin	2D	3D	3D-SFD
OTA	16.87 ± 5.91	>100 *	>100 *
PAT	0.45 ± 0.16	4.93 ± 0.01	>12.5 *

* Highest concentration tested.

## Data Availability

The original contributions presented in the study are included in the article/Supplementary Materials, further inquiries can be directed to the corresponding author.

## Supplementary Materials

The following supporting information can be downloaded at: https://www.mdpi.com/article/10.3390/foods13244167/s1, Figure S1: Negative photomask of the SFD generated using Adobe Illustrator^®^.
