# Supplementary figures and images for: Development of an Easy-To-Use Microfluidic System to Assess Dynamic Exposure to Mycotoxins in 3D Culture Models: Evaluation of Ochratoxin A and Patulin Cytotoxicity
Source: Foods. 2024 Dec 23;13(24):4167. doi: 10.3390/foods13244167 (PMC11675266; doi:10.3390/foods13244167)

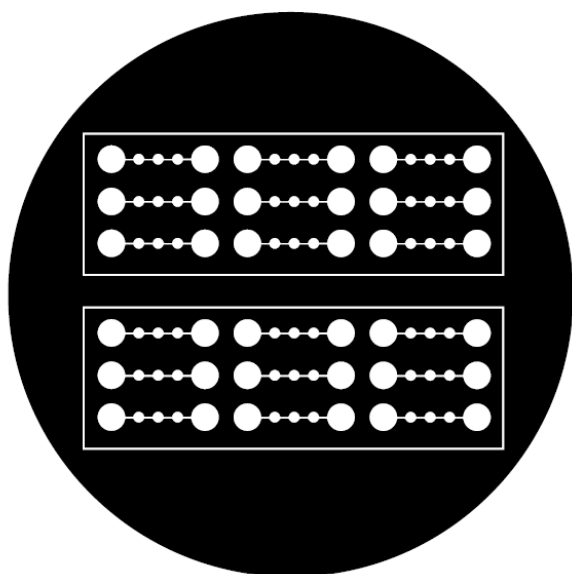

**Figure S1.** Negative photomask of the SFD generated using Adobe Illustrator®.

Supplement: Supplementary file 1 [file foods-13-04167-s001.zip › foods-3312274-supplementary.pdf]
